# Deterioration of Myocardial Global Longitudinal Strain and Its Relationship with Arterial Stiffness in Patients with Cardiac Amyloidosis: A Six-Month Follow-Up

**DOI:** 10.3390/jcm14062078

**Published:** 2025-03-18

**Authors:** Dafni Korela, Emmanouil Foukarakis, Anthοula Plevritaki, Spyros Maragkoudakis, Ioannis Anastasiou, Alexandros Patrianakos, Nikolaos Kapsoritakis, Sophia Koukouraki, Olga Bourogianni, Charalampos Pontikoglou, Maria Psillaki, Helen A. Padadaki, Ioannis Zaganas, Dimitris Samonakis, Eustathios Detorakis, Ioannis Petrakis, Kostas Stylianou, Gregory Chlouverakis, Emmanouil Giannakoudakis, Emmanouil Simantirakis, George Kochiadakis, Maria Marketou

**Affiliations:** 1 Cardiology Department, Venizelion General Hospital of Heraklion, 71409 Heraklion, Greece; koreladafni@hotmail.com (D.K.); mfouk@hotmail.com (E.F.); 2 School of Medicine, University of Crete, 70013 Heraklion, Greece; anthiplevritaki@gmail.com (A.P.); ioannisaanastassiou@yahoo.gr (I.A.); koukour@uoc.gr (S.K.); xpontik@uoc.gr (C.P.); psyllaki@yahoo.gr (M.P.); e.papadaki@uoc.gr (H.A.P.); johnzag@yahoo.com (I.Z.); petrakgia@gmail.com (I.P.); kstylianou@gmai.com (K.S.); gchlouve@uoc.gr (G.C.); esimant@hotmail.com (E.S.); kochiadg@gmail.com (G.K.); 3 Cardiology Department, Heraklion University General Hospital, 71500 Heraklion, Greece; apatrianakos@yahoo.gr; 4 Cardiology Department, General Hospital of Chania ‘St. Andreas’, 73300 Chania, Greece; smaragoudakis79@hotmail.com; 5 Nuclear Medicine Department, Heraklion University General Hospital, 71500 Heraklion, Greece; nikoskapso@hotmail.cm (N.K.); ompourogianni@yahoo.gr (O.B.); 6 Hematology Department, Heraklion University General Hospital, 71500 Heraklion, Greece; 7 Neurology Department, Heraklion University General Hospital, 71500 Heraklion, Greece; 8 Department of Gastroenterology & Hepatology, Heraklion University General Hospital, 71500 Heraklion, Greece; dsamonakis@gmail.com; 9 Radiology Department, Heraklion University General Hospital, 71500 Heraklion, Greece; edetorakis@hotmail.com; 10 Nephrology Department, Heraklion University General Hospital, 71500 Heraklion, Greece; 11 Neurology Department, Venizelion General Hospital of Heraklion, 71500 Heraklion, Greece; emgiannakoudakis@yahoo.com

**Keywords:** amyloidosis, myocardial strain, arterial stiffness

## Abstract

**Background:** Cardiac amyloidosis (CA) is a progressive disorder characterized by amyloid fibril deposition in the heart, leading to heart failure and arrhythmias. Arterial stiffness, assessed by pulse wave velocity (PWV), is recognized as an adverse consequence of amyloidosis, yet its progression and relationship with myocardial dysfunction remain inadequately explored. This study examines the progression of PWV and its potential association with the deterioration of global longitudinal strain (GLS) in CA patients over a 6-month follow-up period. **Methods:** This prospective study enrolled 31 patients who were diagnosed with CA, including both the immunoglobulin light chain (AL) and transthyretin (ATTR) forms. All participants underwent a full echocardiographic study and PWV measurements (carotid-femoral [c-f] and carotid-radial [c-r] PWV) at baseline and 6-month follow-up. Age- and sex-matched individuals with similar cardiovascular risk factors were included as a control group. **Results:** In the CA group, the left ventricular mass index (LVMI) increased significantly from 119.4 ± 52.1 to 124 ± 53.2 g/m^2^ (*p* = 0.002). Both c-f and c-r PWV showed significant increases at the 6-month follow-up (*p* < 0.001 and *p* = 0.005, respectively). The GLS deteriorated significantly from −14 ± 4.4% to −12.8 ± 4.9% (*p* = 0.018). No significant changes were observed in the control group. A weak correlation (r = 0.3; *p* = 0.095) was found between increases in PWV and GLS deterioration. **Conclusions:** Both arterial stiffness and myocardial dysfunction worsen rapidly in CA patients. However, the weak correlation between PWV and GLS suggests that they may evolve through independent mechanisms, necessitating further research to understand their complex interplay in CA.

## 1. Introduction

Amyloidosis is a complex, multisystemic disorder characterized by the deposition of amyloid fibrils in various tissues, resulting from the aggregation of different precursor proteins [1]. Cardiac amyloidosis (CA) is an infiltrative cardiomyopathy caused by the deposition of amyloid fibrils in the heart’s interstitium, formed by misfolded proteins. This leads to biventricular wall hypertrophy, ventricular remodeling, and, eventually, low cardiac output. The disease can affect multiple organs, with cardiac amyloidosis being a significant and often underrecognized cause of heart failure, cardiac arrhythmias, and other cardiovascular complications. Cardiac involvement in systemic amyloidosis is the leading cause of both morbidity and mortality, regardless of the specific amyloid protein involved [2].

The heart is commonly infiltrated by two types of amyloids: immunoglobulin light chain (AL) amyloidosis and transthyretin (ATTR) amyloidosis. Often, these patients are highly symptomatic, experiencing a poor quality of life. In addition to symptoms of heart failure, they frequently suffer from hemodynamic disturbances and/or orthostatic hypotension. Notably, the main causes of morbidity and mortality in amyloidosis are cardiomyopathy and autonomic nervous system neuropathy [3,4,5,6]. Once these conditions emerge, life expectancy is typically 3–5 years for cardiomyopathy and 10 years for neuropathy [6].

Amyloid deposition is also observed in the arterial tree, which can reduce organ perfusion [7]. Additionally, amyloid involvement of the aorta has been reported in several clinical cases [8] and may be associated with increased arterial stiffness [9]. An increased pulse wave velocity (PWV) greater than 9 m/s has been identified in patients with multiple myeloma, suggesting that pre-existing vascular injury may predispose individuals to cardiovascular damage [9]. The infiltration and remodeling of large arteries may correlate with cardiac dysfunction and vasomotor disturbances in the disease. Theoretically, subclinical amyloid infiltration of the aortic wall in cardiac amyloidosis could lead to increased stiffness, impairing left ventricular (LV) function by disrupting arterial–ventricular coupling [10]. Myocardial work which provides a wide-ranging investigation of myocardial function, reflecting both systolic and diastolic performance, has been found to be significantly impaired in patients with cardiac amyloidosis [11,12].

Despite recent diagnostic advancements, the mechanisms behind the effects of amyloid deposition on organ function remain incompletely understood. Gaining better insights into these processes is crucial for developing more effective treatment strategies. This study aims to explore the progression of arterial stiffness in patients with CA, as indicated by PWV, and investigate whether it is associated with the deterioration of the myocardial global longitudinal strain (GLS)—a subclinical systolic index of the left ventricle—over a six-month follow-up period.

## 2. Materials and Methods

### 2.1. Study Population

We conducted a prospective study enrolling 31 consecutive patients with CA. Among these patients, 6 had AL amyloidosis, while the remaining individuals had ATTR amyloidosis. Patients presenting with clinical symptoms of heart failure, along with at least two red flag indicators—spanning clinical, laboratory, electrocardiographic, echocardiographic, and/or cardiac magnetic resonance (CMR) findings—that were suggestive of CA [13] were screened and included in the study. All eligible patients underwent serum-free light chain assays, immunofixation electrophoresis of both serum and urine, and cardiac technetium-99m pyrophosphate ([99mTc]Tc-PYP) bone scintigraphy. CMR imaging and endomyocardial/extracardiac biopsies were conducted in selected cases when deemed necessary by the treating physician. All forms of CA were included in the cases, including both TTR and immunoglobulin light chain (AL) CA. The exclusion criteria included patients under 18 years of age, those with a confirmed diagnosis of other cardiomyopathies (dilated, ischemic, and/or hypertrophic sarcomeric familial cardiomyopathy), heart failure due to severe valvular mitral stenosis or regurgitation, heart failure due to severe aortic regurgitation, or those with confirmed constrictive pericarditis. Additionally, patients lacking available echocardiogram data were excluded from the study. We also excluded patients with a previous diagnosis of epicardial coronary or peripheral artery disease or a past history of an acute coronary event and those with known cardiomyopathies. Patients who altered their medication during the follow-up period or received medication specifically targeting amyloidosis were also excluded. All invited individuals underwent a full clinical examination, electrocardiography, laboratory tests, and echocardiography at baseline and at 6-month follow-up. Age- and sex-matched individuals with no signs of amyloidosis with similar cardiovascular risk factors for atherosclerosis served as controls. The participants’ demographic and past clinical data were collected via direct interviews and measurements or using the institutional electronic medical database.

This study was carried out in accordance with the ethical guidelines of the Declaration of Helsinki of 1975, and the study protocol was approved by our hospital’s Scientific and Ethics Committee and by the hospital administration. All participants signed an informed consent document.

### 2.2. Echocardiographic Study

A standard M-mode and 2-dimensional (2D) echocardiographic test was performed in all participants, in accordance with the recommendations of the American Society of Echocardiography and the European Association of Echocardiography [14,15]. In patients with acute myocarditis, this test was performed during the first 48 h of their admission. The 2D speckle-tracking strain analyses were performed on grayscale images of the left ventricle, using Echopac (GE Medical Systems, Chicago, IL, USA), and the peak GLS was measured.

During the strain analysis, the endocardial border was manually traced at the end-systole, and the width of the region of interest was manually adjusted to include the entire myocardial wall thickness. The Echopac software (GE Medical Systems, Chicago, IL, USA) automatically tracks and accepts segments with good tracking quality and rejects poorly tracked segments. However, the operator is able to manually override computer-generated tracking and accept or reject individual segments based on a visual assessment of the tracking quality.

All GLS measurements were performed blindly by two experienced investigators. The intra-observer variability in GLS measurements was <5%.

### 2.3. Measurement of PWV

All subjects were asked to refrain from caffeine, alcohol, and smoking during the preceding 12 h. The study was carried out between 8:00 and 9:00 AM in a quiet room at 20ߝ22 °C. Height and weight were measured. Subjects were allowed a further 15 min supine rest before baseline measurements. The brachial blood pressure was measured over the brachial artery 3 times at 5 min intervals. The mean of the last 2 measurements was recorded as representative of the brachial blood pressure. After brachial blood pressure, the carotid, femoral, and radial arteries were palpated to find the location of the points with the most pronounced pulse pressure waves. Carotid–femoral (c-f) and carotid–radial (c-r) artery waveforms were measured, and the pwv (Complior SP, Atech Medical, Marsilia, France) was determined. The distances traveled by the pulse waves were assessed in triplicate over the surface of the body with a non-elastic tape measure. The pulse wave transit time was determined from the time delay between the proximal and distal “foot” waveforms.

### 2.4. Statistical Analysis

Summary descriptive statistics are described as mean ± SD or frequencies, as appropriate.

Comparisons of continuous variables between the control and the amyloidosis groups were performed with independent samples *t*-tests, while changes in those variables between baseline and 6 months were evaluated using paired samples *t*-tests. The association between PWV changes and GLS changes was assessed using linear regression techniques and Pearson correlation coefficient. All statistical tests were carried out at the 5% level of significance.

## 3. Results

We included 31 patients with CA, and 31 age- and sex-matched individuals served as the control group. The mean age was 70 ± 14 years, 23 individuals in each group were male, 51% had a history of hypertension, and 48% had a history of diabetes mellitus. The clinical and laboratory data of the participants are summarized in Table 1. An analysis of the data revealed a significant increase in the left ventricular mass index in the amyloidosis group, from 119.4 ± 52.1 g/m^2^ at baseline to 124 ± 53.2 g/m^2^ at six months (*p* = 0.002). No significant changes were observed in the control group after six months (Table 2).

**Figure 1:**
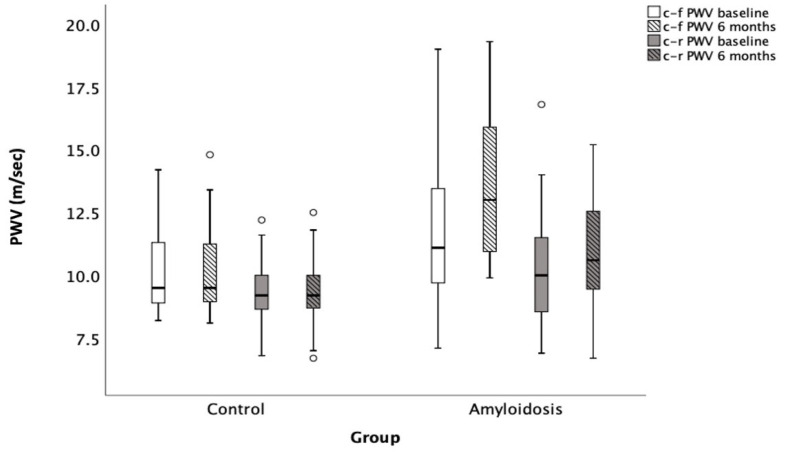
Progression of PWV in patients with cardiac amyloidosis compared to control group over a 6-month follow-up period. c-f PWV: Carotid–femoral pulse wave velocity; c-r PWV: Carotid–radial pulse wave velocity. Open circles denote outliers, i.e., observations that lie more than 1.5 × IQR away from the quartiles.

Notably, patients with cardiac amyloidosis exhibited a significant increase in both their central-to-femoral pulse wave velocity (c-f PWV) and central-to-radial pulse wave velocity (c-r PWV) compared to the control group (Figure 1). Specifically, in the amyloidosis group, the c-f PWV was 11.8 ± 2.7 m/s, compared to 10.2 ± 1.6 m/s in the control group (*p* = 0.008). Similarly, the c-r PWV in the amyloidosis group increased from 9.3 ± 1.2 m/s to 10.7 ± 2.2 m/s (*p* = 0.005), while the c-r PWV in the control group remained at 9.3 ± 1.2 m/s (*p* = 0.041).

Moreover, patients with cardiac amyloidosis showed a significant increase in arterial stiffness over time, especially when compared to healthy controls. This finding may reflect the impact of the disease on both vascular and myocardial function. More specifically, after six months of follow-up, the amyloidosis group demonstrated a significant increase in arterial stiffness, as indicated by a rise in both the c-f PWV and c-r PWV (Figure 1). In particular, the c-r PWV increased from 9.3 ± 1.2 m/s to 10.7 ± 2.2 m/s (*p* = 0.005), while the c-f PWV rose from 11.8 ± 2.7 m/s to 13.4 ± 2.9 m/s (*p* < 0.001) in the amyloidosis group. In contrast, no statistically significant changes were observed in the control group after six months, with the c-r PWV remaining stable at 10.2 ± 1.6 m/s (*p* = NS) and the c-f PWV remaining unchanged at 9.3 ± 1.2 m/s (*p* = NS).

**Figure 2:**
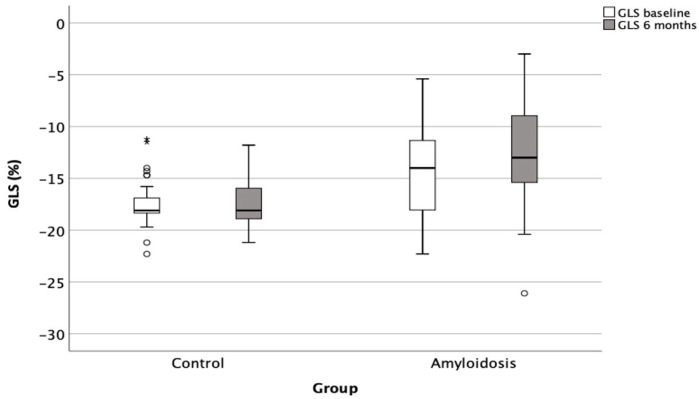
Deterioration of left ventricular GLS in patients with cardiac amyloidosis compared to control group over a 6-month follow-up period. GLS: global longitudinal strain. Open circles denote outliers, i.e., observations that lie more than 1.5 × IQR away from the quartiles. * denote extreme outliers, i.e., observations that lie more than 3 × IQR away from the quartiles. Circles represent the participants, *: <0.05.

Similarly, there was a significant deterioration in the global longitudinal strain (GLS) in the amyloidosis group, from −14 ± 4.4% to −12.8 ± 4.9% (*p* = 0.018), compared to no significant change in the control group (from −17.3 ± 2.3% to −17.3 ± 2.4%; *p* = 0.89) (Figure 2).

We also analyzed the ATTR subgroup, which comprised the majority of our patients, and the findings did not differ significantly from the overall results (the c-f PWV increased from 11.3 ± 2.7 m/s to 13 ± 2.8 m/s, with *p* = 0.04; the c-r PWV rose from 9.8 ± 2.2 m/s to 10.1 ± 2.2 m/s, with *p*= 0.5; and the GLS increased from −14.4 ± 4.4% to −12.1 ± 4.9%, with *p* = 0.2).

**Figure 3:**
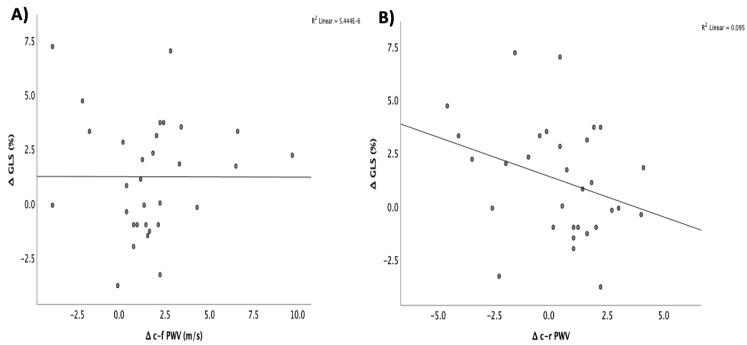
Correlation between the changes in GLS and the changes in (**A**) c-f PWV and (**B**) c-r PWV in patients with cardiac amyloidosis. GLS: global longitudinal strain; c-f PWV: carotid–femoral pulse wave velocity; c-r PWV: carotid–radial pulse wave velocity (circles represent the participants, *: <0.05).

When we examined the relationship between changes in arterial stiffness and the worsening of subclinical systolic dysfunction, as indicated by the GLS, we found only a weak correlation between the increase in c-r PWV and the deterioration in GLS (r = 0.3; *p* = 0.095) (Figure 3). No other statistically significant correlations were observed between the progression of arterial stiffness and other clinical or echocardiographic parameters.

## 4. Discussion

This study is the first to comprehensively examine the progression of arterial stiffness over time and its potential association with the deterioration of myocardial strain in patients with cardiac amyloidosis. We found a notable deterioration in vascular wall properties during the six-month follow-up, as indicated by the increase in aortic stiffness. Similarly, subclinical systolic markers, particularly the left ventricular myocardial strain, also worsened over time. However, the correlation between these two adverse hemodynamic changes was weak, suggesting that the deposition of amyloid in the vasculature and myocardium may not always occur simultaneously or follow a parallel progression. This highlights the complex, independent nature of vascular and myocardial involvement in cardiac amyloidosis. By evaluating both the c-f PWV and c-r PWV, alongside GLS measurements, we provide new insights into how vascular and myocardial dysfunction evolve in this population.

In addition to its cardiac effects, amyloidosis also impacts the vasculature, leading to increased arterial stiffness, which underscores the systemic nature of the disease. Our findings emphasize the progressive nature of both arterial stiffness and myocardial dysfunction. Although the weak correlation between these parameters suggests that their underlying pathophysiological mechanisms may be independent, this warrants further investigation.

PWV has been proposed as a tool of evaluation of vascular injury and pathologic remodeling [13]. Patients with cardiac amyloidosis exhibited significantly increased arterial stiffness, as evidenced by their higher values of c-f PWV and c-r PWV, compared to healthy controls [16,17]. Notably, a previous study has shown that arterial stiffness was an independent predictor of cardiovascular events in patients with amyloidosis, reinforcing the notion that vascular involvement may significantly contribute to the morbidity and mortality associated with this condition [17]. The increase that we found in PWV aligns with previous studies demonstrating that amyloidosis causes the deposition of amyloid fibrils, not only in the heart but also in the vasculature, leading to endothelial dysfunction, vascular remodeling, and stiffening of large arteries [18]. Amyloid deposits in multiple adventitial vessels of the aorta [19], and as its deposits accumulate, they can disrupt the normal elasticity of arterial walls, resulting in higher pulse wave velocities. Our findings, along with those from previous studies, underscore the relevance of monitoring arterial stiffness as a potential marker of disease progression in cardiac amyloidosis. They also further extend this by showing that this increase in stiffness continues over time, suggesting a worsening of vascular involvement as the disease progresses. The association between amyloidosis and vascular stiffness has important clinical implications, as it may contribute to the increased risk of cardiovascular events in these patients.

Additionally, we observed a deterioration in the GLS, indicating subclinical systolic dysfunction, over the follow-up period in the amyloidosis group. Previous studies have shown that the GLS is impaired early in the course of cardiac amyloidosis and can serve as a prognostic marker. It has been shown that the GLS was significantly reduced in patients with cardiac amyloidosis, even when the ejection fraction was preserved, which is a hallmark feature of this disease [20]. Similarly, other studies have reported that the GLS can detect early systolic dysfunction in patients with cardiac amyloidosis before overt clinical manifestations of heart failure develop [21]. The deterioration in the GLS is often observed before traditional measures such as ejection fraction or left ventricular mass changes become clinically apparent, making it an important tool for the early detection of myocardial involvement in amyloidosis [22]. Moreover, in amyloid patients, significant impairments of lobal myocardial constructive work and global myocardial work were observed, which were associated with worse outcomes [23].

The significant deterioration in GLS over the six-month period in patients with cardiac amyloidosis highlights the subclinical systolic dysfunction that is characteristic of cardiac amyloidosis, even in the absence of overt heart failure. In contrast, the control group showed no significant change in GLS, underscoring the progressive nature of myocardial impairment in cardiac amyloidosis. Interestingly, while both the arterial stiffness and GLS worsened over time in the amyloidosis group, there was only a weak correlation between the progression of c-r PWV and the deterioration of GLS. This suggests that while both parameters are influenced by the underlying pathophysiological processes of cardiac amyloidosis, they may not be directly linked in a cause–effect manner. Our results further support the use of the GLS as a sensitive indicator of myocardial dysfunction, providing valuable insight into the subclinical progression of the disease.

Previous data indicated that the end-diastole shear wave velocities of the left ventricle were significantly higher in patients with cardiac amyloidosis, patients with a higher grade of diastolic dysfunction, and elderly volunteers. These findings thus suggest that the speed of naturally induced shear waves may be related to myocardial stiffness [24,25,26,27,28]. However, despite these associations, no strong correlation was found between the progression of arterial stiffness and the worsening of the GLS. This is somewhat unexpected, as one might anticipate a closer relationship between arterial stiffness and myocardial dysfunction, given the known effects of increased arterial stiffness on the myocardial workload and the potential for vascular and myocardial dysfunction to coexist in this disease. We conclude that while both parameters are affected by the disease, their relationship may be more complex than initially hypothesized. In cardiac amyloidosis, the left atrium, like the left ventricle, is adversely affected by amyloid infiltration, resulting in increased size, reduced ejection force, and impaired strain [26]. Notably, the peak LA strain has been shown to correlate with the left ventricular GLS, further emphasizing the interconnected dysfunction of the cardiovascular tree in these patients [26].

Arterial stiffness and myocardial dysfunction likely evolve through different mechanisms in this disease. Myocardial stiffness in amyloidosis is primarily driven by amyloid deposits within the myocardial interstitium, leading to diastolic dysfunction, whereas arterial stiffness is more influenced by amyloid infiltration of the vascular walls, which impacts the elasticity of large arteries [27,28,29]. The observed increases in PWV highlight the progressive nature of vascular changes in this condition, which are known to contribute to adverse cardiovascular outcomes. Previous studies have demonstrated their strong correlation with the risk of developing cardiovascular disease and all-cause mortality [30]. The PWV is also associated with cardiovascular conditions such as impaired diastolic function and altered coronary blood flow and serves as a marker of vascular aging and a tool for estimating cardiovascular risk [30]. In addition, the progression of these two phenomena may occur at different rates. Arterial stiffness may develop earlier in the course of the disease due to the relatively rapid infiltration of the vasculature, while myocardial dysfunction may take longer to manifest in terms of detectable GLS changes. The lack of a strong correlation between the two suggests that other factors, such as the degree of amyloid infiltration, the presence of concomitant cardiovascular risk factors, or the progression of other organ involvement, may influence these parameters independently.

Another noteworthy finding was the significant increase in LVMI in the amyloidosis group. This increase is consistent with the development of myocardial hypertrophy observed in cardiac amyloidosis, which is a result of amyloid deposits in the myocardial wall causing both diastolic and systolic dysfunction [19]. In contrast, no significant changes were observed in the LVMI or other echocardiographic parameters in the control group, which further highlights the disease-specific progression of structural and functional changes in the amyloidosis group.

The observed deterioration in GLS and increase in PWV underscore the importance of early detection and intervention in CA. While our study primarily focused on the progression of these parameters, the clinical implication is that monitoring the GLS and PWV could contribute to a better risk stratification and help identify patients at an early stage of disease progression, potentially before the onset of overt heart failure symptoms or even guiding for more aggressive treatments of the disease. This is particularly relevant because emerging therapies, such as targeting TTR stabilization or AL amyloid fibril formation, are likely to be most effective when initiated early in the disease course. The well-known correlation between the progression of arterial stiffness and myocardial dysfunction also raises interesting therapeutic considerations. It suggests that interventions targeting a single pathway might not be effective and that there is a need for combination therapies in many cases. Given the systemic nature of the disease, combined therapies that address both the vascular and myocardial components of the disease may be more effective than single-target approaches. Moreover, future studies could investigate whether the addition of established cardioprotective therapies could provide incremental benefits in CA patients. Future research should also focus on identifying biomarkers that can predict the response to specific treatments or allow for a more personalized approach.

Our study has several limitations. First, the relatively small sample size limits the generalizability of our findings. The sample size of our prospective observational study was limited due to the rarity of the disease. Larger cohort studies are needed to validate these results and to deepen our understanding of the relationship between arterial stiffness, myocardial dysfunction, and other clinical outcomes in cardiac amyloidosis. Second, although we performed a six-month follow-up, a longer follow-up period would offer better insight into the long-term progression of both arterial stiffness and myocardial dysfunction in this patient population. Additionally, the weak correlation that we observed between changes in arterial stiffness and GLS suggests that other factors—such as the extent of amyloid infiltration, genetic predispositions, or additional biomarkers of disease progression—should be considered in future studies. However, we recognize that smaller sample sizes necessitate caution when interpreting weak correlations or non-significant findings.

Of note, we only studied patients with CA, whereas amyloidosis patients without cardiac affection may differ from our population. An additional limitation of our study is the inclusion of both AL and ATTR amyloidosis, which differ in pathophysiology, clinical presentation, and outcomes. A subgroup analysis would provide pathophysiological insights into the differences between AL and ATTR. However, our small sample size is insufficient to ensure statistical robustness and reliable conclusions. The use of disease-modifying treatments could potentially affect the GLS and arterial stiffness, and future studies should consider the impact of these therapies on cardiovascular parameters in CA patients.

Finally, the lack of a strong correlation between arterial stiffness and GLS raises the possibility that these two parameters may reflect independent markers of disease severity. Future investigations exploring other factors that could influence both myocardial function and vascular health, such as inflammatory markers, oxidative stress, or autonomic dysfunction, may provide a more comprehensive understanding of the mechanisms underlying the clinical manifestations of cardiac amyloidosis. The weak correlation that we observed between changes in PWV and GLS suggests that these two parameters may evolve through largely independent mechanisms. Several factors could explain this discrepancy. Differences in the amyloid burden between myocardial and vascular tissues may play a significant role. Amyloid infiltration can vary considerably, with myocardial deposits primarily impairing contractility and diastolic function, which is reflected in the deterioration of GLS. In contrast, vascular deposits contribute to increased arterial stiffness, as measured by PWV. The extend and distribution of amyloid deposition may differ across these tissues, leading to independent progression of myocardial dysfunction and arterial stiffness. Moreover, autonomic dysfunction is prevalent in CA, which can independently increase the PWV without directly affecting the myocardial strain.

Recent studies have highlighted the potential benefits of sodium-glucose cotransporter 2 inhibitors (SGLT2i) in patients with CA, particularly in reducing the risk of heart failure hospitalizations and improving the clinical outcome [31]. These benefits may be attributed to the cardioprotective and renoprotective effects of SGLT2i and might influence the progression of GLS and PWV in CA patients. Although our study did not specifically examine the impact of SGLT2i on PWV and GLS, future research should consider how these inhibitors might modulate this relationship.

Patients with CA frequently present with a unique set of cardiovascular therapeutic challenges that differ from those seen in other populations. Understanding the pathophysiologies of systolic dysfunction and arterial stiffness could help identify patients who are at higher risk of adverse outcomes, guiding timely interventions. Given the increasing recognition of amyloidosis as a treatable condition, research in this area could significantly impact therapeutic approaches and enhance the quality of care for affected individuals.

## 5. Conclusions

In conclusion, our study demonstrates for the first time that arterial stiffness and myocardial dysfunction both progressively worsen over time at an accelerated rate in patients with cardiac amyloidosis. Both parameters worsen rapidly in these patients, highlighting the need for close monitoring and timely interventions to mitigate their progression. However, the weak correlation between the two suggests that they may evolve through independent pathways. Our findings underscore the complex interplay between arterial stiffness and myocardial function in patients with cardiac amyloidosis and highlight the progressive nature of both vascular and myocardial dysfunction in amyloidosis. Further research is needed to unravel the mechanisms behind these changes and their implications for patient management.

## Figures and Tables

**Table 1:** Clinical and demographic data of participants.

	Patients with CA n = 31	Control Group n = 31	*p*
BMI (kg/m^2^)	25.9 ± 4.3	27.4 ± 2.8	0.24
HR (bpm)	72 ± 7	69 ± 12	0.43
SBP (mmHg)	116 ± 18	130 ± 6	0.001
DBP (mmHg)	73 ± 12	74± 8	0.64
LVEF (%)	50 ± 12	61 ± 6	<0.001
LVMI (g/m^2^)	119.4 ± 52.1	90.4 ± 19.8	0.04
GLS (%)	−14.02 ± 4.4	−17.3 ± 2.4	0.005
c-f PWV (m/s)	11.8 ± 2.7	10.2 ± 2.6	0.007
c-r PWV (m/s)	10.3 ± 2.3	9.3 ± 1.3	0.01
Hemoglobin (g/dL)	13.2 ± 1.9	13.3 ± 1.2	0.12
Glucose (mg/dL)	106 ± 24	100 ± 24	0.1
Creatinine (mg/dL)	1.1 ± 0.5	1 ± 0.4	0.8
Cholesterol (mg/dL)	176 ± 51	172 ± 37	0.8
nt-proBNP (pg/mL)	2244 ± 2852	49 ± 28.2	<0.001

BMI: body mass index; CA: cardiac amyloidosis; c-f PWV: carotid–femoral pulse wave velocity; c-r PWV: carotid–radial pulse wave velocity; DBP: diastolic blood pressure; HR: heart rate; GLS: global longitudinal strain; LVEF: left ventricular ejection fraction; LVMI: left ventricular mass index; nt-proBNP: N-terminal prohormone of brain natriuretic peptide; SBP: systolic blood pressure.

**Table 2:** Changes in clinical and echocardiographic parameters at the 6-month follow-up.

	Patients with CA n = 31			Control Group n = 31		
	Baseline	6 Months	*p*	Baseline	6 Months	*p*
BMI (kg/m^2^)	27.4 ± 2.8	27 ± 5.1	0.5	25.9 ± 4.3	25.6 ± 4.3	0.6
Troponine I (ng/mL)	268 ± 435	324 ± 488	0.7	4.5 ± 6.5	5.1 ± 6.8	0.8
LVEF (%)	61 ± 6	61.6 ± 5.3	0.9	50 ± 12	48.8 ± 4.7	0.057
LVMI (g/m^2^)	90.4 ± 19.8	90.5 ± 19.9	0.9	119.4 ± 52.1	124 ± 53.2	0.02
GLS (%)	−17.3 ± 2.4	−17.2 ± 2.2	0.9	−14.02 ± 4.4	−12.8 ± 4.9	0.018
c-f PWV (m/s)	10.2 ± 2.6	10.1 ± 2.4	0.8	11.8 ± 2.7	13.4 ± 2.9	0.03
c-r PWV (m/s)	9.3 ± 1.3	9.4 ± 1.2	0.8	10.3 ± 2.3	10.8 ± 2.5	0.3

BMI: body mass index; CA: cardiac amyloidosis; GLS: global longitudinal strain; c-f PWV: carotid–femoral pulse wave velocity; c-r PWV: carotid–radial pulse wave velocity; LVEF: left ventricular ejection fraction; LVMI: left ventricular mass index.

## Data Availability

The data presented in this study are available on request from the corresponding author. The data are not publicly available due to institutional policies requiring a data-sharing agreement.

## Abbreviations

The following abbreviations are used in this manuscript:

LV	left ventricle
c-f PWV	carotid–femoral pulse wave velocity
c-r PWV	carotid–radial pulse wave velocity
GLS	global longitudinal strain
